# Corneal remodelling and topography following biological inlay implantation with combined crosslinking in a rabbit model

**DOI:** 10.1038/s41598-019-39617-0

**Published:** 2019-03-14

**Authors:** Iben Bach Damgaard, Yu-Chi Liu, Andri Kartasasmita Riau, Ericia Pei Wen Teo, Min Li Tey, Chan Lwin Nyein, Jodhbir Singh Mehta

**Affiliations:** 1 Department of Ophthalmology, Aarhus University Hospital, Aarhus, Denmark; 2 Tissue Engineering and Stem Cell Group, Singapore Eye Research Institute, Singapore, Singapore; 3 School of Materials Science and Engineering, Nanyang Technological University, Singapore, Singapore; 4 Singapore National Eye Centre, Singapore, Singapore; 5 Ophthalmology and Visual Sciences Academic Clinical Programme, Duke-NUS Graduate Medical School, Singapore, Singapore; 6 Yong Loo Lin School of Medicine, National University of Singapore, Singapore, Singapore

## Abstract

Implantation of biological corneal inlays, derived from small incision lenticule extraction, may be a feasible method for surgical management of refractive and corneal diseases. However, the refractive outcome is dependent on stromal remodelling of both the inlay and recipient stroma. This study aimed to investigate the refractive changes and tissue responses following implantation of 2.5-mm biological inlays with or without corneal collagen crosslinking (CXL) in a rabbit model. Prior to implantation, rotational rheometry demonstrated an almost two-fold increase in corneal stiffness after CXL. After implantation, haze gradually subsided in the CXL-treated inlays (p = 0.001), whereas the untreated inlays preserved their clarity (p = 0.75). *In-vivo* confocal microscopy revealed reduced keratocyte cell count at the interface of the CXL inlays at week 8. Following initial steepening, regression was observed in anterior mean curvature from week 1 to 12, being most prominent for the non-CXL subgroups (non-CXL: −12.3 ± 2.6D vs CXL: −2.3 ± 4.4D at 90 μm depth, p = 0.03; non-CXL: −12.4 ± 8.0D vs CXL: −5.0 ± 4.0D at 120 μm depth, p = 0.22). Immunohistochemical analysis revealed comparable tissue responses in CXL and untreated subgroups. Our findings suggest that CXL of biological inlays may reduce the time before refractive stabilization, but longer postoperative steroid treatment is necessary in order to reduce postoperative haze.

## Introduction

Presbyopia is an age-related physiological condition with gradual loss of accommodation, that causes an inability to focus at near distance^1^. Corneal inlays for correction of presbyopia may be implanted under a flap or into a stromal pocket to increase the depth of field (Kamra inlay, AcuFocus), alter the refractive index of the central cornea (Presbia Flexivue Microlens, Presbia Cooperatief), or reshape the corneal surface (Raindrop Near Vision inlay, ReVision Optics)^2^. A major advantage of corneal inlays is the reversibility of the procedure, as they can be removed if the patients are dissatisfied with their visual outcome^3,4^. However, corneal synthetic inlays may cause biocompatibility related problems with the risk of anterior stromal ulceration and keratolysis, inlay-edge deposits, and interface inflammation^5–8^. Hence, biological inlays may serve as a favourable substitution for synthetic inlays, and offer the advantage of better biocompatibility due to unobstructed passage of oxygen and nutrients^9^.

Small incision lenticule extraction (SMILE) for myopia and myopic astigmatism involves the creation of an intrastromal lenticule using the VisuMax 500-kHz femtosecond laser (Carl Zeiss Meditec, Jena, Germany)^10,11^. The plano-convex shaped stromal lenticule is extracted through a small incision to flatten the anterior surface. The lenticule is normally discarded after surgery but may potentially be used for tissue additive surgery or for tissue volume restoration^12–22^. Meniscus and doughnut shaped biological inlays have previously been used for anterior corneal curvature flattening in patients with keratoconus^16,18,20^ while plano-convex shaped lenticules have been used for corneal perforations^23^. Although there is a significant difference between using biological tissue for management of pathological corneas and refractive disorders, a few studies have also reported successful corneal curvature steepening in patients with hyperopia^15,19^, presbyopia^14^ and aphakia^17^ following intrastromal implantation of biological inlays.

For surgical management of presbyopia, the biological inlays may act as a shape changing inlay, that creates a central hyperprolate contour for near and intermediate vision^9^, similar to what is seen following implantation of the Raindrop inlay^9,14^. The inlays may be removed and replaced according to the refractive status of the patient, as previous studies using rabbits and non-human primates have shown the reversibility of the procedure in terms of refraction and corneal thickness^21,22,24^. The diameter of the biological inlay can be customized by trephining the centre of the lenticule, as the average lenticule diameter in myopic SMILE is between 5 and 7 mm.

We have previously demonstrated in non-human primates that lenticule implantation of the central 3-mm of a −3D SMILE derived lenticule effectively caused a hyperprolate shape change^9^. However, regression of the corneal steepening was observed in the initial 4 months after implantation, possibly explained by some degree of stromal and epithelial remodelling. Corneal collagen crosslinking (CXL) of the biological inlays prior to implantation may be an option to reduce their flexibility to natural stromal remodelling after implantation, driving the host stroma to conform to the shape of the inlays instead. Hence, the aim of this study was to examine the corneal topography and stromal remodulation following implantation of SMILE-derived CXL treated and non-CXL treated biological inlays.

## Methods

### Rotational rheometry and corneal collagen crosslinking

The biomechanical strength of 7 rabbits lenticules (3 CXL treated and 4 controls) and 7 cryopreserved human SMILE-derived lenticules (3 CXL treated and 4 controls) were measured *ex vivo* using a rotational rheometer (MCR 502; Anton-Paar, Graz, Austria). One control human and rabbit lenticule were used as sacrificial specimens for dynamic strain sweep test. Pairwise comparisons were performed using CXL treated and control lenticules with similar refractive power. The human lenticules had been cryopreserved for 2 weeks at −80 °C as described previously^25,26^. These lenticules were thawed at 37 °C, rinsed in PBS, and stored in Optisol BS (Bausch&Lomb Surgical, Irvine, CA) until CXL.

**Figure 1:**
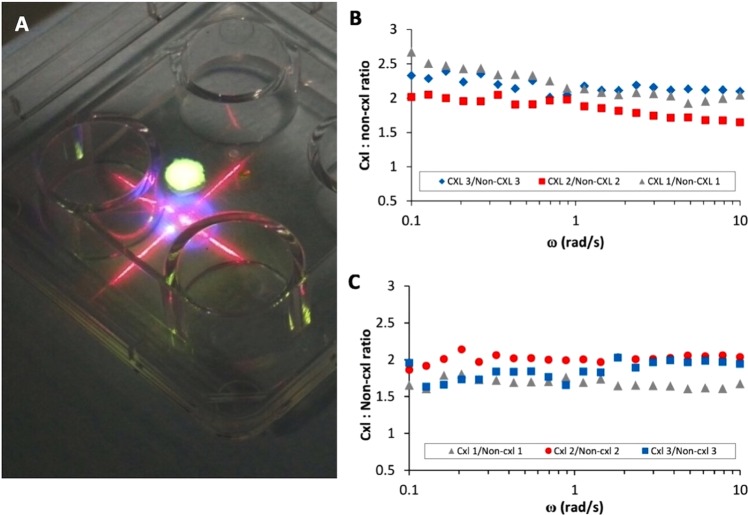
Comparison of biomechanical strength between non-CXL and CXL lenticules assessed by rheometry. (**A**) Corneal collagen crosslinking of a rabbit lenticule. A central 2.5-mm button was trephined after CXL and used for implantation. Frequency dependent crosslinked lenticule/non-crosslinked lenticule G’ ratio for (**B**) human lenticules and (**C**) rabbit lenticules.

The human and rabbit lenticules that underwent CXL treatment were soaked in Riboflavin dye (vitamin B2) for four minutes. After positioning on a firm surface, the excess riboflavin dye was removed with a sponge. Accelerated crosslinking with UV-A radiation was performed using an Avedro CXL system (Waltham, MA) at a wavelength of 365 μm (Fig. 1A). The illumination process was conducted at 30 mW/cm^2^ for four minutes, delivering a total dose of 7.20 J/cm^2^.

Immediately following CXL, the lenticules were subjected to rheometry similar to a previously published protocol^27^. The lenticule was placed and centred under a parallel plate geometry, and wet with Optisol BS solution to avoid moisture evaporation. Temperature was maintained at 25 °C for all measurements. The top plate was then lowered slowly until a contact (loading not exceeding 0.1N) was made with the lenticule. The sample was allowed to relax for 1 minute before the top plate was lowered further with micron precision to 5% more than the thickness of the lenticule that had been measured with anterior segment optical coherence tomography (AS-OCT, RTVue Fourier-Domain OCT; Optovue, Fremont, CA). The specimen was then left to relax for another 1 minute before the measurement was initiated. During this time the stress relaxed to zero and the resulting equilibrium thickness of lenticule was considered as the loading gap. In order to obtain reliable measurements in the linear viscoelastic regime, a dynamic strain sweep test at a frequency of 1 rad/s from 0.5–5% strain was performed on the sacrificial lenticules to determine the linear strain range to apply to the subsequent measurements. Because the response was relatively linear in the tested strain range of 0.5–5% (Fig. S1A,C), the subsequent measurements were performed at strain amplitude of 1% within a frequency window of 0.1–10 rad/s. Finally, the Young’s modulus of the lenticule was calculated as three times of the viscoelastic plateau of G’ modulus based on the theory of linear viscoelascocity^28^.

### Study animals and experimental groups

Thirteen 12–15 weeks old New Zealand female white rabbits weighing 3–4 kg were used for autologous implantation. The right eye of each rabbit underwent SMILE for tissue harvesting while the left eye underwent tissue implantation. The rabbits were randomly allocated into four groups: non-CXL inlays implanted at 90 μm depth (non-CXL-90 group, n = 3 eyes) and 120 μm depth (non-CXL-120 group, n = 3 eyes) and CXL treated inlays implanted at 90 μm depth (CXL-90 group, n = 3 eyes) and 120 μm depth (CXL-120 group, n = 3 eyes). One rabbit (n = 2 eyes) served as an untreated control.

The research protocol was approved by the Institutional Animal Care and Use Committee (IACUC) of SingHealth, Singapore, and conducted in accordance of the ARVO statement for the Use of Animals in Ophthalmic and Vision Research. During pre- and postoperative examination and surgical intervention, the rabbits were anesthetized by an intramuscular injection of xylazine hydrochloride (5 mg/kg, Troy Laboratories, Smithfield, Australia) and ketamine hydrochloride (50 mg/kg, Parnell Laboratories, Alexandria, Australia). The rabbits were euthanized under anaesthesia by an overdose intracardiac injection of sodium pentobarbital (Jurox, Rutherford, Australia).

### Surgical procedure

Myopic SMILE correction was performed in the right eye of the rabbits using the VisuMax 500-kHz femtosecond laser as described previously^11^. The following settings were used: 110 μm cap thickness, 7.5 mm cap diameter, 6.5 mm lenticule diameter, 63 μm maximum lenticule thickness, 10 μm minimum lenticule thickness, and −3D spherical power. The laser power was 200 nJ, with and a spot distance and tracking spacing of 3 μm/3 μm for the lenticule and cap, and 2 μm/μm for the lenticule side cut. A seibel spatula (Rhein Medical Inc. Petersburg, FL) was used to open the superior incision and locate the anterior and posterior lenticule surface. The remaining tissue bridges were broken with a SMILE dissector (Asico, AE2403 LLC), and the lenticule extracted with a pair of forceps. The stromal pocket was irrigated with balanced salt solution via a 24 gauge-cannula.

Six lenticules underwent CXL performed using same procedure and energy settings as in the above section. The CXL treated (n = 6) and non-CXL treated (n = 6) SMILE-derived lenticules were spread out with a surgical sponge, and 2.5-mm inlays were trephined from the lenticule centre.

An intrastromal flocket was created in the left eye of the rabbits using the VisuMax femtosecond laser^29^. A 7.5 mm cap diameter and 330-degree hinge cut was used, thereby giving a 30° superior incision for lenticule implantation. The biological inlay was inserted in the flocket and a lamellar dissector used to spread and centrally align the tissue over the pupil centre. One experienced surgeon (JSM) performed all surgical procedures. A subconjunctival injection of dexamethasone sodium phosphate (40 mg/ml; Shin Poong Pharmaceutical, South Korea) and gentamicin (40 mg/ml; Shin Poong Pharmaceutical) was given at the end of the procedure. Postoperative regime included 0.3% Tobramycin (Alcon, Fort Worth, TX) and 0.1% Dexamethasone acetate (Allergan, Irvine, CA) four times daily for 1 week.

### Clinical evaluation

The preoperative and 1, 2, 4, 6, 8, and 12-week postoperative examination included bio-microscopy (Zoom Slit Lamp NS-2D, Tokyo, Japan), AS-OCT, and corneal topography (Visante Omni, Carl Zeiss Meditec, Jena, Germany). Spherical aberrations (SA, Z4^0^) and the root mean square of total higher order aberrations (RMS of total HOA, 3^rd^ to 7^th^ Zernike order) were evaluated by Zernike wavefront analysis (ATLAS 9000, Carl Zeiss Meditec) in a 6.00-mm pupil zone under mydriatic conditions. Inlay clarity was graded on a scale of 0 to 4 (0: completely clear to 4: completely obscured)^30^. Central corneal thickness (CCT), inlay thickness, and anterior lamellar thickness were acquired from the high-resolution AS-OCT images using ImageJ (http://imagej.nih.gov/ij/, National Institutes of Health, Bethesda, MD, USA). The median of three measurements was used for statistical analysis. To quantify the stromal intensity on OCT images, we measured the Mean Gray Value (MGV) of the inlay and the recipient stroma in a corresponding area. The ratio MGV(inlay)/MGV(recipient) was then calculated for the CXL treated (n = 6) and non-CXL treated (n = 6) inlays.

### *In-Vivo* Confocal Microscopy

*In-vivo* confocal microscopy (IVCM) was performed pre- and 4, 8, and 12 weeks postoperatively (HRT3; Heidelberg Engineering GmbH, Heidelberg, Germany). The stromal keratocyte reflectivity was evaluated by selecting 6 micrographs from the interface, and from the planes anterior and posterior to the implanted inlay (n = 2 for each) and semi-quantifying the mean grey value (MGV) of reflectivity using Image J, as described before^31,32^. The keratocyte cell count for each included rabbit was calculated by the mean of 2 anterior and 2 posterior micrographs of the inlay and was performed by a single masked observer (E.T).

### Histology and immunohistochemistry

After 12-weeks, the rabbits were euthanized and the inlay implanted eyes excised. The corneas were embedded in optimal cutting temperature compound (Leica Microsystems, Nussloch, Germany), stored at −80 °C, and cryosectioned in 8 μm thickness (Microm HM550 cryostat, Walldorf, Germany). The tissue underwent hematoxylin-eosin (H&E) staining before examination under a light microscope (Axioplan 2, Carl Zeiss, Oberkochen, Germany). Immunofluorescent staining was performed as previously described in detail^9,29^. Incubation in antibodies against alpha-smooth muscle actin (α-SMA; Agilent, Santa Clara, CA), fibronectin (Millipore, Burlington, MA), tenascin-C (Abcam, Cambridge, UK), CD11b (BD Pharmingen, Franklin Lakes, NJ), and heat shock protein 47 (HSP47; Enzo Life Sciences, Switzerland) were performed overnight at 4 °C. After washing with 0.01 M phosphate buffered saline (PBS; Life Technologies), incubation with Alexa Flour 488-conjugated secondary antibody (Jackson ImmunoRes Lab, West Grove, USA) was performed at room temperature for one hour. To detect cell apoptosis, fluorescence-based terminal deoxynucleotidyl transferase dUTP nick end labeling (TUNEL) assay was performed according to the manufacturer’s instructions (Click-iT TUNEL Alexa-Fluor Imaging Assay, Thermo Fisher Scientific, Waltham, MA). Subsequently, all lenticules were mounted with Fluoroshield mounting medium containing DAPI (4′,6-diamidino-2-phenylindole, Santa Cruz Biotechnology, Santa Cruz, CA), and viewed under a fluorescence microscope (Axioplan 2).

### Statistical analysis

Statistical analysis was performed in Stata (version 13; STATACorp, College Station, TX) and GraphPad Prism (v6.0 h; GraphPad Software Inc., La Jolla, CA). Student unpaired t-test was used for comparison between groups, while paired t-test was used to evaluate the change between two time points. Mean values and standard deviations are reported. A p-value < 0.05 was considered statistically significant. The datasets generated during the current study are available from the corresponding author on reasonable request.

## Results

### Rotational rheometry

Rotational rheometry was conducted in both human and rabbit lenticules before continuing with implantation in rabbits. The dynamic strain sweep tests indicated that both lenticules with and without CXL were within the linear viscoelastic regime at 0.5–5% strain at 1 rad/s frequency (Fig. S1A,C). The CXL procedure yielded a consistent G’ modulus improvement of approximately two-fold compared to non-treated control lenticules in human lenticules (Figs 1B and S1B) and in rabbit lenticules (Figs 1C and S1D). Young’s modulus of the human lenticules ranged from 111–2540 Pa after CXL treatment and from 54–1210 Pa without CXL treatment (Table 1). The Young’s modulus of the rabbit lenticules ranged from 198–218 Pa after CXL and from 105–118 Pa without CXL treatment. The average enhancement of viscoelastic properties following the CXL procedure was 1.93 ± 0.25 times and 1.89 ± 0.19 times in human and rabbit lenticules, respectively.

**Table 1:** Young’s modulus of lenticules and improvement ratio between crosslinked and non-crosslinked lenticules.

Sample	Young’s modulus (Pa)		CXL: Non-CXL Young’s modulus ratio	Mean ratio
	CXL	Non-CXL		
Rabbit 1	198	118	1.67	
Rabbit 2	214	105	2.04	1.89 ± 0.19
Rabbit 3	218	112	1.95	
Human 1	2540	1210	2.10	
Human 2	182	111	1.65	1.93 ± 0.25
Human 3	111	54	2.04	

### Slit lamp biomicroscopy

**Figure 2:**
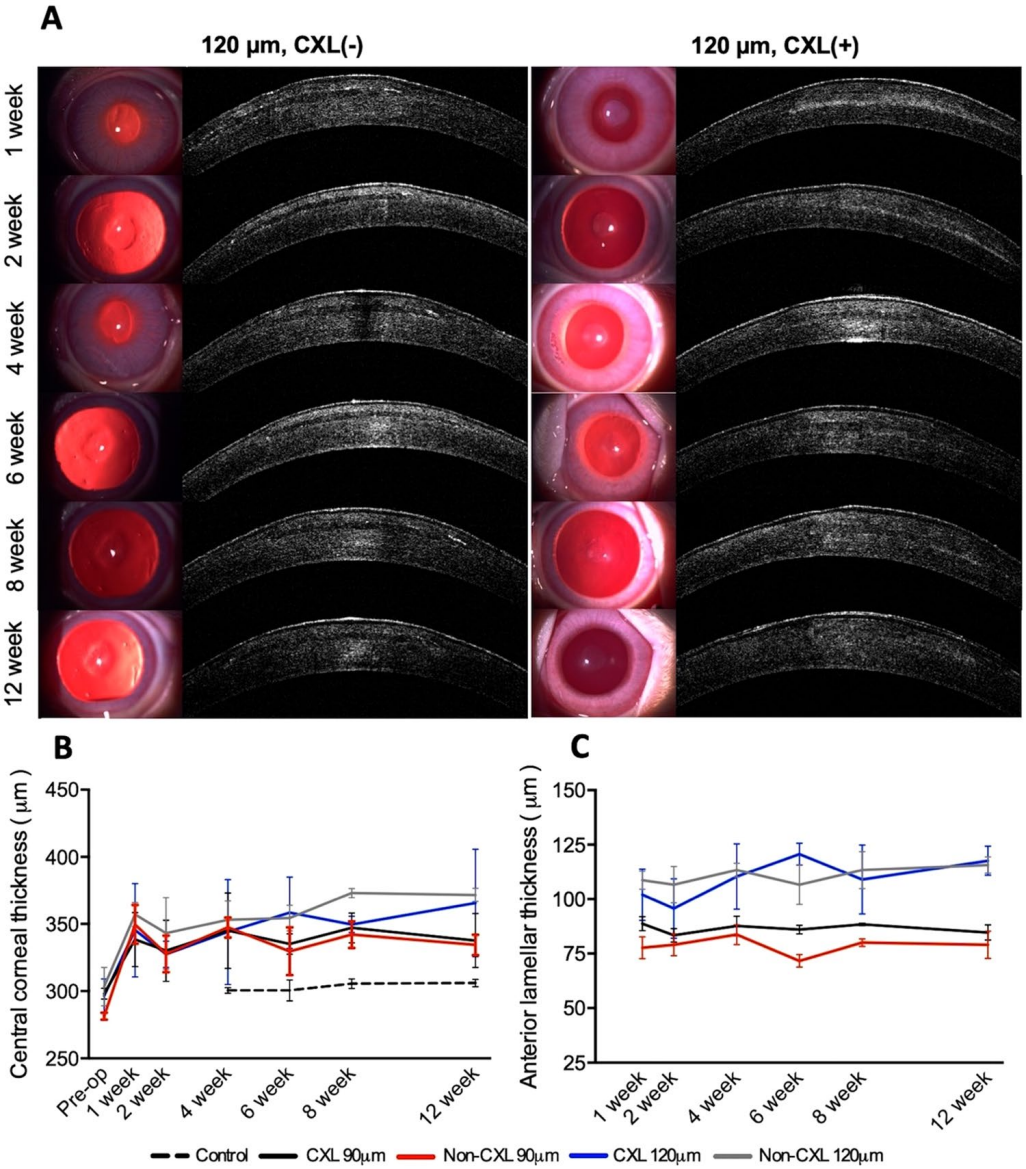
Slit lamp observation and anterior segment-optical coherence tomography (AS-OCT) of corneas following implantation of CXL and untreated inlays. (**A**) Slit-lamp biomicroscopy and anterior segment optical coherence tomography (ASOCT). Left column: Untreated inlays. The inlay preserved their stromal clarity during the postoperative period. Right column: CXL treated inlays. The postoperative stromal haze progressively improved from week 1 to week 12. (**B**) The development in the average central corneal thickness and (**C**) the anterior lamellar thickness. Bars represents standard deviations.

Mild corneal oedema was observed around the inlay the first week after implantation (Fig. 2A). The non-CXL inlays remained clear 1-week postoperatively and throughout the remaining the follow up period (clarity score 1 week: 0.92 ± 0.20, 12 week: 0.75 ± 0.27, p = 0.75, Fig. S2A). The inlays were still distinguishable from the recipient cornea 12 weeks postoperatively, although the edge of the inlays was no longer clearly visible. Haze was observed in all CXL treated inlays after implantation (1 week: 3.17 ± 0.75), that gradually resolved after 8 weeks (0.92 ± 0.20) and during the remaining follow up period (12 week: 0.83 ± 0.26, p = 0.001).

### Anterior segment optical coherence tomography (AS-OCT)

At 1-week, the AS-OCT showed easily distinguishable non-CXL treated inlays, whereas the contour of the CXL treated inlays were more difficult to locate (Fig. 2A). When evaluating the mean grey value (MGV), the CXL treated inlays (n = 6) were denser than their stromal surroundings at week 1 and 2, with a MGV_inlay_/MGV_recipient_ ratio of 1.34 ± 0.47 and 1.33 ± 0.44, respectively (Fig. S2B). The OCT intensity gradually decreased to a ratio of 0.98 ± 0.20 at 12 week examination. The non-CXL treated inlays (n = 6) were less dense than their stromal surroundings with a MGV ratio < 1 during the entire follow up period (Fig. S2B). No significant difference was seen in the MGV ratio between CXL and non-CXL treated inlays 12 weeks postoperatively (p = 0.06).

The change in CCT over time, evaluated using AS-OCT, is found in Fig. 2B. The central corneal thickness increased from 298.0 ± 4.0 μm to 337.7 ± 20.1 μm, from 296.3 ± 12.7 μm to 365.7 ± 40.1 μm, from 281.3 ± 2.5 μm to 334.0 ± 7.6 μm, and from 303.3 ± 14.2 μm to 371.7 ± 4.9 μm at 12 weeks postoperatively, for the CXL-90, CXL-120, non-CXL-90, and non-CXL-120 groups, respectively. The anterior lamellar thickness averaged 84.7 ± 3.5 μm, 117.7 ± 6.7 μm, 79.0 ± 6.2 μm, and 115.7 ± 3.8 μm, for the CXL-90, CXL-120, non-CXL-90, and non-CXL-120 groups at 12 weeks, respectively (Fig. 2C). In all groups, the lamellar thickness remained stable after implantation (p > 0.052), with a significant difference between the 90 and 120 μm groups (p < 0.001). At 12-weeks, the average inlay thickness was 59.3 ± 6.1 μm, 59.3 ± 5.5 μm, 59.0 ± 0.0 μm, and 61.3 ± 0.6 μm for the CXL-90, CXL-120, non-CXL-90, and non-CXL-120 subgroups, respectively (Fig. S3).

### Corneal topography and aberrations

**Figure 3:**
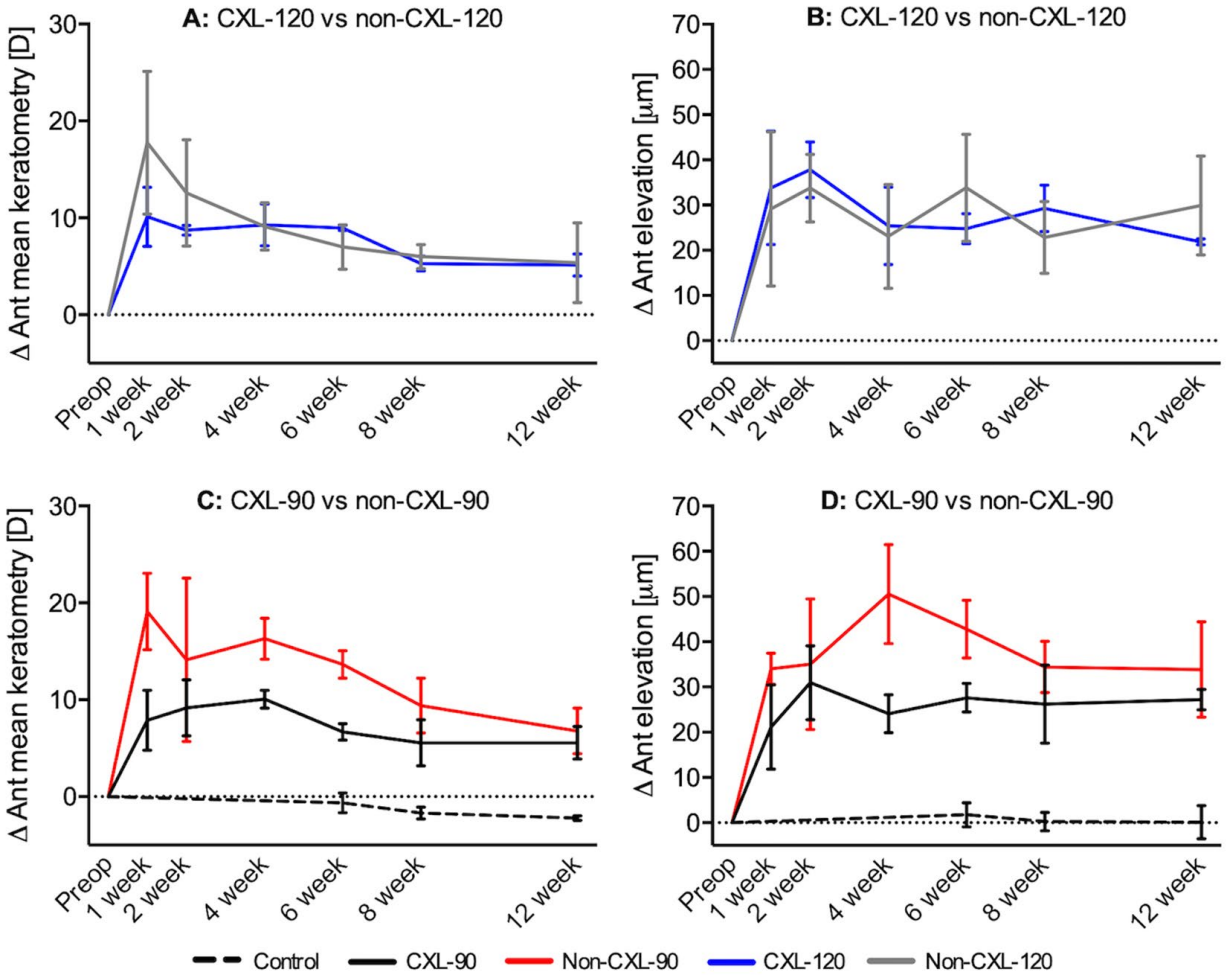
Refractive changes following implantation of non-CXL and CXL inlays at different corneal depths. The average change in (**A**,**C**) anterior mean curvature and (**B**,**D**) anterior elevation at different time points during postoperative follow up. Bars represents standard deviations.

The changes in anterior mean curvature and anterior elevation at different time points are summarized in Table 2 and Fig. 3. At 1 week, the anterior mean curvature increased after implantation in all four groups (p < 0.042). At 12 weeks, the average increase in anterior mean curvature was 5.6 ± 1.7D, 5.1 ± 1.1D, 6.8 ± 2.4D, and 5.4 ± 4.1D for the CXL-90, CXL-120, non-CXL-90, and non-CXL-120 subgroups, respectively. Regression in the corneal steepening was observed in both CXL and non-CXL treated groups from week 1 to week 12, with a tendency towards less regression in the CXL treated groups (Table 2, CXL-90 vs. non-CXL-90: p = 0.03. CXL-120 vs. non-CXL-120: p = 0.22). The anterior elevation significantly increased after implantation in the CXL-120 group and non-CXL-90 group (p < 0.042), but not in the CXL-90 and non-CXL-120 group (p > 0.05, Table 2, Fig. 3B,D). At 12 weeks, the average increase in anterior elevation was 27.2 ± 2.3 μm, 22.0 ± 0.7 μm, 33.8 ± 11.6 μm, and 29.9 ± 8.0 μm for the CXL-90, CXL-120, non-CXL-90, and non-CXL-120 subgroups respectively.

**Table 2:** Average increase in anterior mean curvature and anterior elevation following implantation, mean ± standard deviation.

	Δ Anterior mean curvature [D]				Δ Anterior elevation [µm]			
	90 µm depth		120 µm depth		90 µm depth		120 µm depth	
	Non-CXL n = 3	CXL n = 3	Non-CXL N = 3	CXL n = 3	Non-CXL n = 3	CXL n = 3	Non-CXL n = 3	CXL n = 3
1 week	19.1 ± 4.0	7.9 ± 3.1	17.8 ± 7.4	10.1 ± 3.0	34.0 ± 3.5	21.1 ± 9.3	29.2 ± 17.1	34 ± 12.5
2 week	14.1 ± 8.4	9.2 ± 2.9	12.6 ± 5.5	8.7 ± 0.5	35.0 ± 14.4	30.9 ± 8.2	33.7 ± 7.5	38 ± 6.2
4 week	16.3 ± 2.1	10.1 ± 0.9	9.1 ± 2.4	9.3 ± 2.1	51.5 ± 10.9	24.1 ± 4.2	23.1 ± 11.5	25 ± 8.6
6 week	13.6 ± 1.4	6.7 ± 0.8	7.0 ± 2.3	9.0 ± 0.3	42.7 ± 6.7	27.6 ± 3.1	33.8 ± 11.8	25 ± 3.3
8 week	9.4 ± 2.8	5.6 ± 2.4	6.0 ± 1.2	5.3 ± 0.8	34.4 ± 6.7	26.2 ± 0.6	22.8 ± 8.0	29.0 ± 5.1
12 week	6.8 ± 2.4	5.6 ± 1.7	5.4 ± 4.1	5.1 ± 1.1	33.8 ± 11.6	27.2 ± 2.3	29.9 ± 8.0	22.0 ± 0.7
12 week-1week	−12.3 ± 2.6^*^	−2.3 ± 4.4^*^	−12.4 ± 8.0	−5.0 ± 4.0	−0.2 ± 10.3	6.1 ± 7.9	0.8 ± 6.9	−11.9 ± 10.8

*Significant difference between non-CXL and CXL groups.

The RMS of total HOA increased in all groups with an average of 2.09 ± 1.22 μm, 2.42 ± 0.04 μm, 2.81 ± 0.56 μm and 3.21 ± 0.48 μm in the CXL-90, CXL-120, non-CXL-90 and non-CXL-120 groups at week 12. The average change in SA was −1.68 ± 0.59 μm, −1.92 ± 0.24 μm, 0.10 ± 0.59 μm and −0.50 ± 0.15 μm in the CXL-90, CXL-120, non-CXL-90 and non-CXL-120 groups, with a significant difference between the CXL and untreated subgroups (p < 0.022).

### *In Vivo* Confocal Microscopy

**Figure 4:**
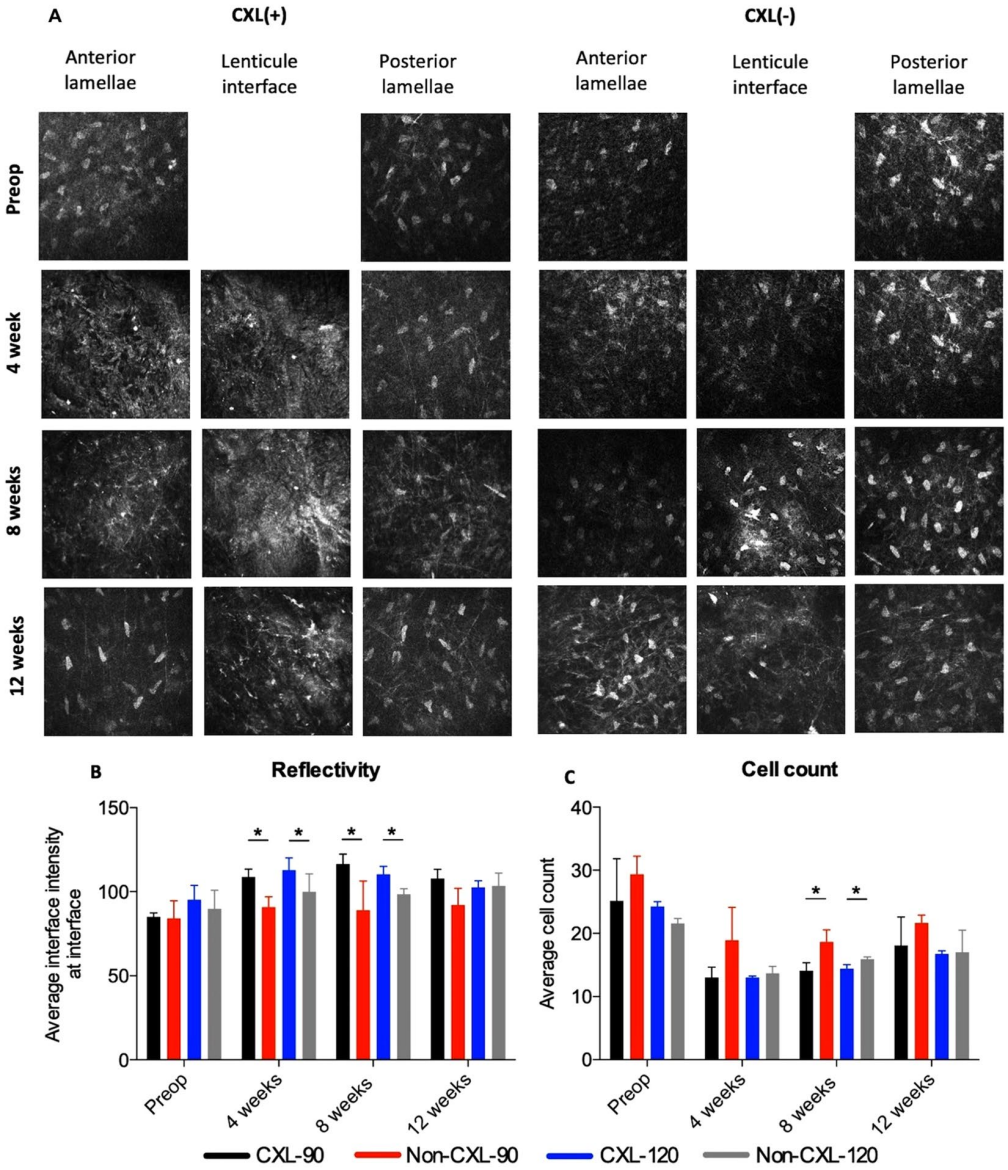
*In vivo* confocal microscopy of corneal stroma following implantation of non-CXL and CXL inlays. (**A**) Representative *in vivo* confocal micrographs of the anterior stroma, inlay interface and posterior stroma. (**B**) The average intensity measured in mean gray value (MGV) using a semi-automated tracking program (ImageJ). (**C**) Average keratocyte cell count evaluated by the mean of four representative images of the interface. Bars represent standard deviations. *Significant difference, p < 0.05.

*In-vivo* confocal micrographs (Fig. 4A) showed hyper-reflective bands in the CXL treated stromal inlays and low keratocyte density at week 4 and 8. Small hyper-reflective particles were seen at week 4 and 8, possibly attributed to inflammatory cells or keratocyte apoptotic bodies, which had almost resolved at the 12-week examination. Haze was observed in the anterior corneal stroma at week 4 and 8 in the CXL-treated groups, which gradually resolved. When comparing the CXL and untreated inlays, higher interface intensity was seen in the CXL treated inlays at week 4 (p < 0.03) and week 8 (p < 0.007, Fig. 4B). This difference was not observed 12 weeks postoperatively. As seen by the average cell count, there was a tendency towards fewer keratocyte cells in the CXL treated inlays than in the corresponding untreated inlays (Fig. 4C, p < 0.02 at 8 weeks).

### Immunohistochemistry

**Figure 5:**
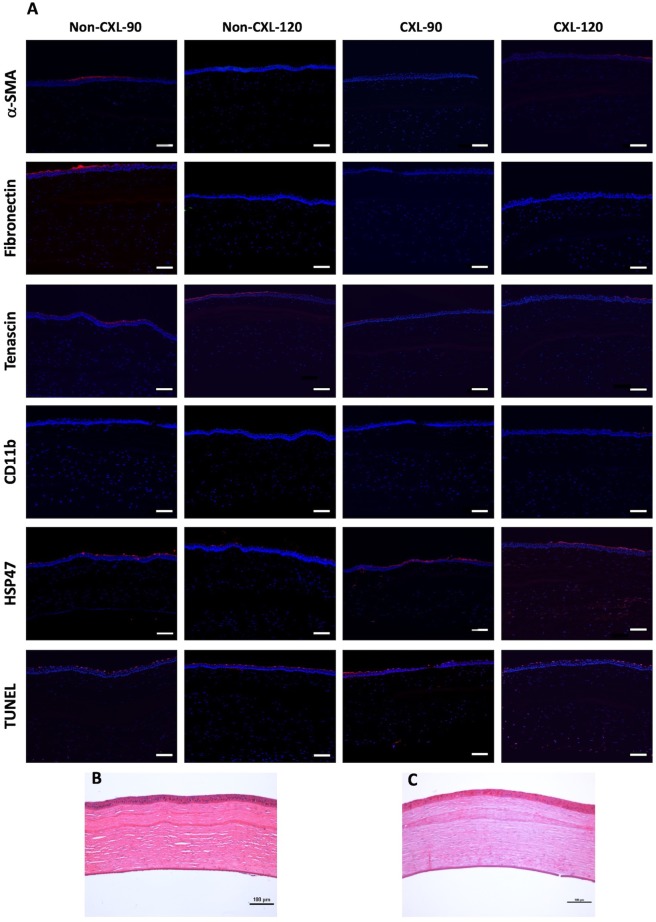
Tissue responses following implantation of non-CXL and CXL inlays in rabbit corneas. (**A**) Immunohistochemistry assays of α-SMA, fibronectin, tenascin, CD11b, heat shock protein 47 (HSP47), and TUNEL 12 weeks postoperatively. Nuclei are counterstained using DAPI (blue). All images were captured at x100 magnification; scale bars represent 100 µm. (B + C) Representative hematoxylin-eosin staining images for CXL treated inlays at (**B**) 90 μm and (**C**) 120 μm depth. Original magnification: 100x. Scale bars represent 100 µm.

Fibroblast activation, indicated by expression of α-SMA, was absent in all samples (Fig. 5A). Fibronectin, tenascin, and CD11b, were also undetectable in both the CXL treated and untreated groups. There was a low expression of HSP47 and a few TUNEL-positive cells in the CXL groups. The HSP47 and TUNEL positive staining was primarily located in the surrounding recipient tissue of the CXL subgroups and not in the biological inlays. HSP47 and TUNEL markers were undetectable in the non-CXL treated subgroups.

### Histological analysis

The recipient corneal stroma showed normal ultrastructure and arrangement of the collagen fibres after implantation (Fig. 5B,C). At 12 weeks postoperatively, the edge of the CXL treated inlays were clearly distinguishable from the surrounding tissue and the lenticule stroma was devoid of keratocytes. No apparent signs of inflammatory cells or fibrotic tissue were seen around the CXL treated inlays.

## Discussion

This study demonstrated that intrastromal implantation of a 2.5-mm diameter inlay, derived from a −3D SMILE lenticule (maximum lenticule thickness of 63 μm), successfully steepened the corneal curvature, with an average increase in the anterior mean curvature between 5.1 and 6.8D after 12 weeks. When evaluating the anterior mean curvature, CXL of the inlays seems to cause less regression in the postoperative period when compared with untreated controls. However, moderate haze was observed in the CXL treated inlays that gradually subsided the first 8 weeks. Furthermore, *in-vivo* confocal microscopy revealed higher interface intensity in the CXL treated inlays compared with the untreated inlays at week 4 and 8.

Corneal collagen crosslinking relies on a photochemical reaction between riboflavin and ultraviolet A that increases the amount chemical bonds between the stromal collagen fibres and strengthen the corneal tissue^33^. Before *in vivo* implantation, we used rotational rheometry to establish if the CXL protocol had been effective. The frequency sweep test (intermediate strain value of 1%) showed an obvious quantitative improvement of the G’ modulus across all frequencies following CXL, with an approximately two-fold improvement of G’ modulus compared to non-treated control lenticules in both species. The variation in Young’s modulus in the human lenticules was attributed to the different thickness of the lenticules that we obtained from patients, where the thicker lenticules (from higher SMILE correction) resulted in greater Young’s modulus. We performed accelerated corneal crosslinking using a total irradiance of 7.2 J/cm^2^ to ensure the treatment was effective^34^. However, lenticule thinning was observed following CXL. It is likely that a reduced total irradiance would be just as efficient to stiffen the biological inlays which remains to be investigated.

Corneal haze is a common complication following CXL that decreases the transparency of the tissue and thus would limit the visual benefit of a biological inlay^35^. Haze was present in the CXL treated inlays, that decreased from grade 3.17 ± 0.75 at 1 week to grade 0.83 ± 0.26 at 12 weeks (Fig. S2A). Furthermore, the IVCM revealed keratocyte apoptosis seen by lower keratocyte cell count in CXL treated inlays than in untreated inlays that is consistent with previous studies of CXL in human corneas^36,37^. Increased keratocyte intensity was observed following CXL, likely due to the presence of activated keratocytes^37^ that may contribute to the CXL-associated corneal haze^38^. The postoperative regime included topical 0.3% tobramycin and 0.1% dexamethasone four times daily for 1 week in all groups. However, the current study suggests that a low dose topical dexamethasone is recommended for a longer postoperative period, to reduce the postoperative haze formation^39^.

The H&E staining revealed absence of keratocytes in the stromal inlays of the CXL treated groups, with a regular arrangement of the collagen fibres. The acellullar stromal arrangement was expected as the riboflavin acts as a photosensitizer that increases the absorption of UVA and causes keratocyte damage^40,41^.

On AS-OCT, the CXL treated inlays were generally thinner (CXL-90: 49.3 ± 5.1 μm vs. non-CXL-90: 70.7 ± 2.5 μm) and with higher OCT intensity (mean MGV ratio of 1.34 ± 0.47 vs. 0.84 ± 0.14) than the untreated inlays at 1 week, suggesting that the CXL treatment of the inlays was effective^42,43^. The difference in inlay thickness between CXL and untreated inlays equalized at 12 weeks to an average thickness between 59.0 ± 0.0 and 61.3 ± 0.6 μm, possibly due to stromal remodelling of both CXL and untreated inlays. In our previous study of non-human primates, the implantation of a 65 μm SMILE derived biological inlay^9^ seemed to cause corneal steepening comparable to what is seen following implantation of a 32 μm synthetic Raindrop inlay^44^. Regression of the corneal steepening was observed until 4 months postoperatively before the corneal topography stabilized. Epithelial remodelling, commonly seen after refractive surgery, may explain some of the regression^45^. However, the need of a thicker biological inlay to achieve the same amount corneal steepening suggests that remodelling of the stromal inlay also occurs in the postoperative period^9^.

In our previous study of non-human primates, implantation of 3.0-mm biological inlays in 120 μm depth caused an increase in the simulated keratometry of 1.8–2.3D after 6 months^9^. In this study, using a rabbit model, the corneal steepening effect was more noticeable following implantation, with an anterior mean curvature increase of 5.4 ± 4.1D at 12 weeks (non-CXL-120 group). The slightly smaller inlay (2.5-mm diameter) used in the current study and a higher percentage of stromal volume expansion in rabbits may contribute to some of the difference. The more likely explanation, however, is the undeveloped Bowman’s membrane in rabbits that would normally counteract the steepening effect of an intrastromal inlay^46^. The CXL treated inlays seemed to cause less corneal steepening in the initial weeks following implantation, when compared with untreated inlays (Fig. 3A,C). Furthermore, implantation in the more superficial layers seemed to induce more corneal steepening than implantation in the deeper corneal layers, although non-significant (non-CXL-90: 6.8 ± 2.4D vs. non-CXL-120: 5.4 ± 41D, p = 0.63). These results are, however, in agreement with previous studies of biological lenticule implantation *ex vivo* in human donor corneas and *in vivo* in non-human primates^47,48^. We observed an increase in the RMS of total HOA while the SA became more negative following biological implantation, consistent with previous reported higher-order aberrations after implantation of artificial^44^ and biological inlays^49^.

Before commencement of a clinical trial, it was important to evaluate if CXL treatment of the corneal inlays would reduce the postoperative stromal remodelling and time until refractive stabilization. Further research is needed to optimize the CXL protocol, and the postoperative topical treatment in order to reduce the early postoperative haze. We were able to show an almost two-fold increase in stromal stiffness with the current CXL protocol, but reduced radiant exposure may be used in future studies. We conducted this pilot study, to evaluate the effect of CXL on the refractive outcome and acknowledge the limitation of a small sample size due to strict regulations of use of animals in scientific research. A small sample size undeniably increases the risk of type 2 errors and must be taken into consideration when interpreting the statistical outcome^50^.

In conclusion, this study demonstrated that lenticule implantation of a 2.5-mm biological inlay steepens the anterior curvature with corneal stabilization after 8 weeks. Despite the limitations of a small sample size, we did see a tendency towards less regression for CXL treated inlays when compared with corresponding untreated inlays. However, haze following implantation was more severe in CXL treated inlays, and increased keratocyte activity was seen up to 8 weeks postoperatively. Prolonged postoperative treatment with topical steroid is recommended if CXL of inlays is performed prior to implantation.

## Supplementary information

Supplementary material

## References

[1] Strenk SA (1999). Age-related changes in human ciliary muscle and lens: a magnetic resonance imaging study. Invest. Ophthalmol. Vis. Sci..

[2] Konstantopoulos A, Mehta JS (2015). Surgical compensation of presbyopia with corneal inlays. Expert Rev. Med. Devices.

[3] Dexl AK (2015). Long-term outcomes after monocular corneal inlay implantation for the surgical compensation of presbyopia. J. Cataract Refract. Surg..

[4] Jalali S, Aus der Au W, Shaarawy T (2016). AcuFocus Corneal Inlay to Correct Presbyopia Using Femto-LASIK. One Year Results of a Prospective Cohort Study. Klin. Monbl. Augenheilkd..

[5] Ismail MM (2006). Correction of hyperopia by intracorneal lenses. Two-year follow-up. J. Cataract Refract. Surg..

[6] Mulet ME, Alio JL, Knorz MC (2009). Hydrogel Intracorneal Inlays for the Correction of Hyperopia. Outcomes and Complications after 5 Years of Follow-up. Ophthalmology.

[7] Duignan ES (2016). Corneal inlay implantation complicated by infectious keratitis. Br. J. Ophthalmol..

[8] Ong HS, Chan AS, Yau CW, Mehta JS (2018). Corneal Inlays for Presbyopia Explanted Due to Corneal Haze. J. Refract. Surg..

[9] Liu Y-C (2018). Biological corneal inlay for presbyopia derived from small incision lenticule extraction (SMILE). Sci. Rep..

[10] Shah R, Shah S, Sengupta S (2011). Results of small incision lenticule extraction: All-in-one femtosecond laser refractive surgery. J. Cataract Refract. Surg..

[11] Sekundo W, Kunert KS, Blum M (2011). Small incision corneal refractive surgery using the small incision lenticule extraction (SMILE) procedure for the correction of myopia and myopic astigmatism: results of a 6 month prospective study. Br. J. Ophthalmol..

[12] Li M, Zhao F, Li M, Knorz MC, Zhou X (2018). Treatment of Corneal Ectasia by Implantation of an Allogenic Corneal Lenticule. J. Refract. Surg..

[13] Bhandari V, Ganesh S, Brar S, Pandey R (2016). Application of the SMILE-Derived Glued Lenticule Patch Graft in Microperforations and Partial-Thickness Corneal Defects. Cornea.

[14] Jacob S (2017). Preliminary Evidence of Successful Near Vision Enhancement With a New Technique: PrEsbyopic Allogenic Refractive Lenticule (PEARL) Corneal Inlay Using a SMILE Lenticule. J. Refract. Surg..

[15] Sun L (2015). The Safety and Predictability of Implanting Autologous Lenticule Obtained by SMILE for Hyperopia. J. Refract. Surg..

[16] Mastropasqua L, Nubile M, Salgari N, Mastropasqua R (2018). Femtosecond Laser–Assisted Stromal Lenticule Addition Keratoplasty for the Treatment of Advanced Keratoconus: A Preliminary Study. J. Refract. Surg..

[17] Pradhan KR (2013). Femtosecond Laser-Assisted Keyhole Endokeratophakia: Correction of Hyperopia by Implantation of an Allogeneic Lenticule Obtained by SMILE From a Myopic Donor. J. Refract. Surg..

[18] Ganesh S, Brar S (2015). Femtosecond Intrastromal Lenticular Implantation Combined With Accelerated Collagen Cross-Linking for the Treatment of Keratoconus—Initial Clinical Result in 6 Eyes. Cornea.

[19] Ganesh S, Brar S, Rao PA (2014). Cryopreservation of extracted corneal lenticules after small incision lenticule extraction for potential use in human subjects. Cornea.

[20] Jacob S (2018). Corneal Allogenic Intrastromal Ring Segments (CAIRS) Combined With Corneal Cross-linking for Keratoconus. J. Refract. Surg..

[21] Angunawela RI, Riau AK, Chaurasia SS, Tan DT, Mehta JS (2012). Refractive lenticule re-implantation after myopic ReLEx: a feasibility study of stromal restoration after refractive surgery in a rabbit model. Invest. Ophthalmol. Vis. Sci..

[22] Riau, A. K. *et al*. Reversible Femtosecond Laser-Assisted Myopia Correction: A Non-Human Primate Study of Lenticule Re-Implantation after Refractive Lenticule Extraction. *PLoS One* **8** (2013).. 10.1371/journal.pone.0067058 PMC3691223 23826194

[23] Wu F, Jin X, Xu Y, Yang Y (2015). Treatment of corneal perforation with lenticules from small incision lenticule extraction surgery: a preliminary study of 6 patients. Cornea.

[24] Sun Y (2016). Reversible Femtosecond Laser-Assisted Endokeratophakia Using Cryopreserved Allogeneic Corneal Lenticule. J. Refract. Surg..

[25] Mohamed-Noriegaarim K (2011). Cornea lenticule viability and structural integrity after refractive lenticule extraction (ReLEx) and cryopreservation. Mol. Vis..

[26] Liu Y-C (2017). Corneal lenticule storage before reimplantation. Mol. Vis..

[27] Aslanides IM (2016). Assessment of UVA-Riboflavin Corneal Cross-Linking Using Small Amplitude Oscillatory Shear Measurements. Investig. Opthalmology Vis. Sci..

[28] Macosko, C. W. *Rheology: Principles*, *Measurements*, *and Application*s (1994).

[29] Konstantopoulos A (2017). Early wound healing and refractive response of different pocket configurations following presbyopic inlay implantation. PLoS One.

[30] Fantes FE (1990). Wound Healing After Excimer Laser Keratomileusis (Photorefractive Keratectomy) in Monkeys. Arch. Ophthalmol..

[31] Liu Y-C (2016). Wound healing profiles of hyperopic-small incision lenticule extraction (SMILE). Sci. Rep..

[32] Liu Y-C, Teo EPW, Lwin NC, Yam GHF, Mehta JS (2016). Early Corneal Wound Healing and Inflammatory Responses After SMILE: Comparison of the Effects of Different Refractive Corrections and Surgical Experiences. J. Refract. Surg..

[33] Wollensak G, Spoerl E, Seiler T (2003). Riboflavin/ultraviolet-a-induced collagen crosslinking for the treatment of keratoconus. Am. J. Ophthalmol..

[34] Woo JH (2017). Conventional Versus Accelerated Collagen Cross-Linking for Keratoconus: A Comparison of Visual, Refractive, Topographic and Biomechanical Outcomes. Open Ophthalmol. J..

[35] Gutiérrez R, Lopez I, Villa-Collar C, González-Méijome JM (2012). Corneal Transparency After Cross-linking for Keratoconus: 1-Year Follow-up. J. Refract. Surg..

[36] Mazzotta C (2015). *In Vivo* Confocal Microscopy after Corneal Collagen Crosslinking. Ocul. Surf..

[37] Aminifard M-N, Khallaghi H, Mohammadi M, Jafarzadeh R (2015). Comparison of corneal keratocytes before and after corneal collagen cross-linking in keratoconus patients. Int. Ophthalmol..

[38] Dhawan S, Rao K, Natrajan S (2011). Complications of corneal collagen cross-linking. J. Ophthalmol..

[39] Xu, L. *et al*. Clinical Study of Mitomycin C in Reducing Haze Formation After Ultraviolet A/Riboflavin Crosslinking for Keratoconus. *Eye Contact Lens*. (2017). 10.1097/ICL.0000000000000422 28945648

[40] Wollensak G, Spoerl E, Wilsch M, Seiler T (2004). Keratocyte apoptosis after corneal collagen cross-linking using riboflavin/UVA treatment. Cornea.

[41] Song W (2017). The comparative safety of genipin versus UVA-riboflavin crosslinking of rabbit corneas. Mol. Vis..

[42] Kymionis GD (2009). Intraoperative Pachymetric Measurements during Corneal Collagen Cross-Linking with Riboflavin and Ultraviolet A Irradiation. Ophthalmology.

[43] Rechichi M (2016). Intraoperative OCT Pachymetry in Patients Undergoing Dextran-Free Riboflavin UVA Accelerated Corneal Collagen Crosslinking. Curr. Eye Res..

[44] Whang W-J, Yoo Y-S, Joo C-K, Yoon G (2017). Changes in Keratometric Values and Corneal High Order Aberrations After Hydrogel Inlay Implantation. Am. J. Ophthalmol..

[45] Reinstein DZ, Archer TJ, Gobbe M, Kanellopoulos AJ, Asimellis G (2014). Rate of change of curvature of the corneal stromal surface drives epithelial compensatory changes and remodeling. J. Refract. Surg..

[46] Hayashi S, Osawa T, Tohyama K (2002). Comparative observations on corneas, with special reference to bowman’s layer and descemet’s membrane in mammals and amphibians. J. Morphol..

[47] Damgaard IB, Ivarsen A, Hjortdal J (2018). Biological lenticule implantation for correction of hyperopia: an *ex-vivo* study in human corneas. J. Refract. Surg..

[48] Williams GP (2018). Hyperopic refractive correction by LASIK, SMILE or lenticule reimplantation in a non-human primate model. PLoS One.

[49] Liu Y-C (2018). Higher-Order-Aberrations Following Hyperopia Treatment: Small Incision Lenticule Extraction, Laser-Assisted *In Situ* Keratomileusis and Lenticule Implantation. Transl. Vis. Sci. Technol..

[50] De Winter JCF (2013). Using the Student’s t-Test with Extremely Small Sample Sizes. Practical Assessment, Research & Evaluation..

