# Supplementary material for: Corneal remodelling and topography following biological inlay implantation with combined crosslinking in a rabbit model
Source: Sci Rep. 2019 Mar 14;9:4479. doi: 10.1038/s41598-019-39617-0 (PMC6418097; doi:10.1038/s41598-019-39617-0)
Supplement: Supplementary file 1 — Supplementary material [file 41598_2019_39617_MOESM1_ESM.pdf]

Corneal remodelling and topography following biological inlay implantation with combined crosslinking in a rabbit model

Iben Bach Damgaard, MD [1,2]
Yu-Chi Liu, MD, MCI [2,4,5]
Andri Kartasasmita Riau, PhD [2,3]
Ericia Pei Wen Teo [2]
Min Li Tey [2,6]
Chan Lwin Nyein [2]
Jodhbir Singh Mehta, FRCS [2,3,4,5] *

[1] Department of Ophthalmology, Aarhus University Hospital, Aarhus, Denmark
[2] Tissue Engineering and Stem Cell Group, Singapore Eye Research Institute, Singapore
[3] School of Materials Science and Engineering, Nanyang Technological University, Singapore
[4] Singapore National Eye Centre, Singapore
[5] Ophthalmology and Visual Sciences Academic Clinical Programme, Duke-NUS Graduate Medical School, Singapore
[6] Yong Loo Lin School of Medicine, National University of Singapore

Word count: 4309 words

Financial support: This research was supported by the Singapore National Research Foundation under its Translational and Clinical Research (TCR) Programme (NMRC/TCR/1021-SERI/2013) and administered by the Singapore Ministry of Health's National Medical Research Council. Supported in part by grants from The Synoptik Foundation and Fight for Sight, Denmark.

Competing interests: Dr. Mehta is a consultant for Ziemer and Carl Zeiss Meditec, Inc. The remaining authors have no conflicts of interests.

Correspondence:
Assoc Prof Jodhbir S. Mehta
Singapore Eye Research Institute,
The Academia, 20 College Road, Discovery Tower Level 6, Singapore 169856
Tel: +65-6227-7255; Fax: +65-6227-7290
E-mail: jodmehta@gmail.com

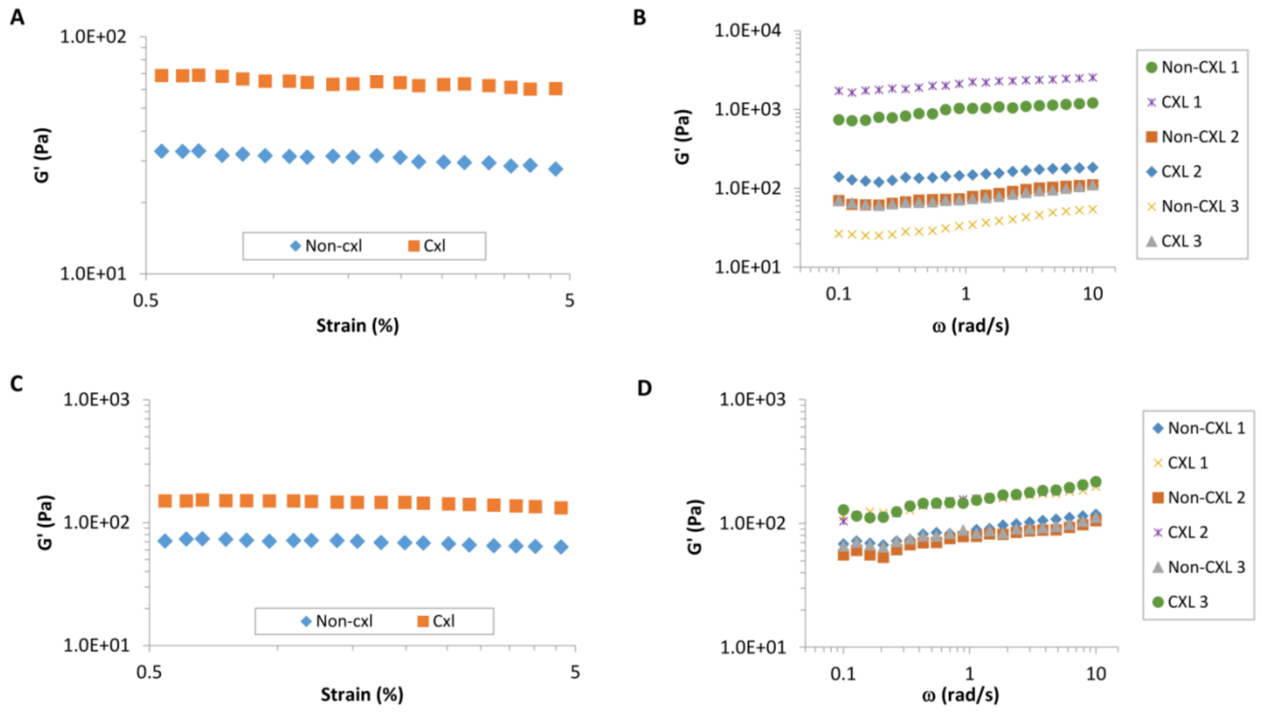

Supplementary Figure S1. Dynamic strain sweep and comparison of frequency dependent G' of crosslinked and non-crosslinked lenticules. (A) Dynamic strain sweep of crosslinked and non-crosslinked human lenticules performed at 1 rad/s and room temperature. (B) G' of paired crosslinked and non-crosslinked human lenticules performed at 1% strain and room temperature. (C) Similar to human lenticules, dynamic strain sweep of rabbit lenticules was also performed at 1 rad/s and room temperature. (D) G' of paired crosslinked and non-crosslinked rabbit lenticules.

A

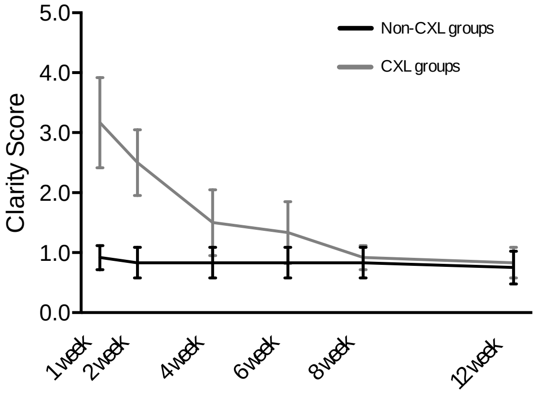

	Non-CXL n=6	CXL n=6
**1 week**	0.92 ±0.20	3.17 ±0.75
**2 week**	0.83 ±0.26	2.50 ±0.55
**4 week**	0.83 ±0.26	1.50 ±0.55
**6 week**	0.83 ±0.26	1.33 ±0.52
**8 week**	0.83 ±0.26	0.92 ±0.20
**12 week**	0.75 ±0.27	0.83 ±0.26

B

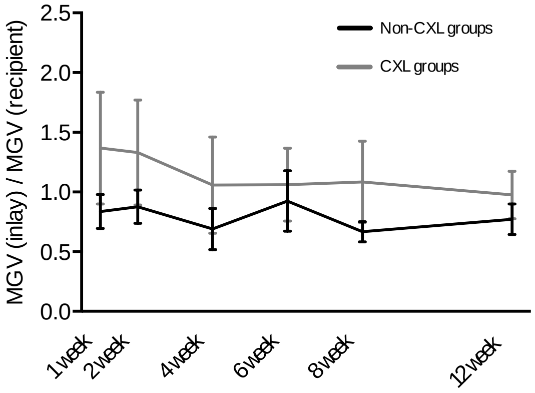

	Non-CXL n=6	CXL n=6
**1 week**	0.84 ±0.14	1.34 ±0.47
**2 week**	0.88 ±0.14	1.33 ±0.44
**4 week**	0.69 ±0.17	1.06 ±0.40
**6 week**	0.93 ±0.25	1.06 ±0.31
**8 week**	0.67 ±0.08	1.09 ±0.34
**12 week**	0.77 ±0.13	0.98 ±0.20

Supplementary Figure S2: Clarity of non-CXL and CXL inlays following intrastromal implantation. (A) Clarity Score for the CXL treated and untreated inlays, evaluated by biomicroscopy. 0: None, 4: Severe. (B) The ratio of the mean gray value (MGV_{inlay} / $MGV_{recipient}$) for the CXL treated (CXL-90 and CXL-120) and untreated groups (non-CXL-90 and non-CXL-120). The MGV, ranging from 0 to 255, was measured using 32-bit grey scale OCT images and the ImageJ software. No difference in MGV ratio was seen between the CXL treated and untreated inlays at 12-week examination (p=0.06).

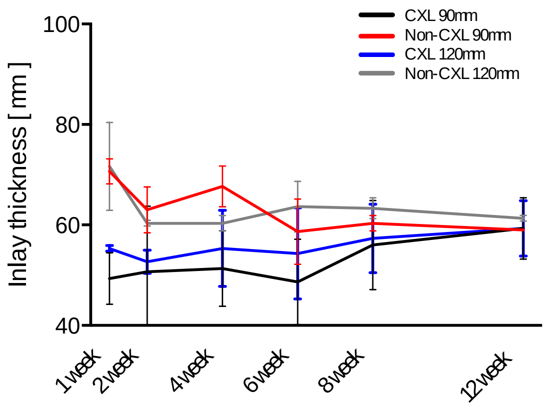

	90 µm depth		120 µm depth	
	**Non-CXL n=3**	**CXL n=3**	**Non-CXL n=3**	**CXL n=3**
**1 week**	70.7±2.5	49.3±5.1	71.7±8.7	55.3±0.6
**2 week**	63.0±4.6	50.7±13.1	60.3±0.6	52.7±2.3
**4 week**	67.7±4.0	51.3±7.5	60.3±1.5	55.3±7.6
**6 week**	58.7±6.5	48.7±8.5	63.7±5.0	54.3±9.1
**8 week**	60.3±1.5	56.0±8.9	63.3±2.1	57.3±6.8
**12 week**	59.0±0.0	59.3±6.1	61.3±0.6	59.3±5.5

Supplementary Figure S3: The average inlay thickness acquired using anterior segment optical coherence tomography and the ImageJ software.
